# Comparison of cardiac function between single left ventricle and tricuspid atresia: assessment using echocardiography combined with computational fluid dynamics

**DOI:** 10.3389/fped.2023.1159342

**Published:** 2023-04-17

**Authors:** Li-Jun Chen, Lan-Ping Wu, Lei-Sheng Zhao, Zhi-Fang Zhang, Jin-Long Liu, Wen-Jing Hong, Shu-Wen Zhong, Sheng-Fang Bao, Jing Yang, Yu-Qi Zhang

**Affiliations:** ^1^ Department of Pediatric Cardiology, Shanghai Children’s Medical Center, School of Medicine, Shanghai Jiao Tong University, Shanghai, China; ^2^ Institute of Pediatric Translational Medicine, Shanghai Children’s Medical Center, School of Medicine, Shanghai Jiao Tong University, Shanghai, China; ^3^ Department of Ultrasound, Jiaxing University Affiliated Women and Children Hospital, Jiaxing, China

**Keywords:** functional single left ventricle, tricuspid atresia, single left ventricle, cardiac function, three-dimensional speckle tracking echocardiography (3DSTE), computational fluid dynamics—CFD

## Abstract

Patients with single left ventricle (SLV) and tricuspid atresia (TA) have impaired systolic and diastolic function. However, there are few comparative studies among patients with SLV, TA and children without heart disease. The current study includes 15 children in each group. The parameters measured by two-dimensional echocardiography, three-dimensional speckle tracking echocardiography (3DSTE), and vortexes calculated by computational fluid dynamics were compared among these three groups. Twist is best correlated with ejection fraction measured by 3DSTE. Twist, torsion, apical rotation, average radial strain, peak velocity of systolic wave in left lateral wall by tissue Doppler imaging (sL), and myocardial performance index are better in the TA group than those in the SLV group. sL by tissue Doppler imaging in the TA group are even higher than those in the Control group. In patients with SLV, blood flow spreads out in a fan-shaped manner and forms two small vortices. In the TA group, the main vortex is similar to the one in a normal LV chamber, but smaller. The vortex rings during diastolic phase are incomplete in the SLV and TA groups. In summary, patients with SLV or TA have impaired systolic and diastolic function. Patients with SLV had poorer cardiac function than those with TA due to less compensation and more disordered streamline. Twist may be good indicator for LV function.

## Introduction

Single left ventricle (SLV) and tricuspid atresia (TA) are two common subtypes of functional single left ventricle (FSLV), a kind of complex cyanotic congenital heart disease with single or systemic left ventricle (1). Cardiac dysfunction is common in these patients during the midterm and long-term follow-up after the Fontan procedure (2).

The impact of ventricular morphology on ventricular function and clinical outcomes are controversial. Only limited studies were focused on the characteristics of different subtypes of FSLV (3–6). Altered ventricular geometry such as increased sphericity and extension of the apex resulting from overload may consequently increase ventricular function (7). Geometry, structure, and systolic function affect its filling and even diastolic function, as noted by Rösner et al. (8). Compared with SLV, TA appears to have a more ellipsoid shape of the LV. Therefore, we propose that TAs have better cardiac function than SLVs do.

Echocardiography is a reliable and convenient clinical assessment tool for ventricular function measurement (9). It has good correlations with cardiac magnetic resonance (CMR) (10) and, therefore, can be applied in FSLV (11). Computational fluid dynamics (CFD) is a new and useful method that can help visualize the fluid field inside the cardiac chamber (12). This study utilized echocardiography combined with the CFD method to assess and compare the cardiac function in children with SLV or TA, and establish good parameters for cardiac function analysis.

## Methods

### Subjects

Fifteen patients with SLV and 15 with TA who were diagnosed using echocardiography and confirmed by surgery from August 2015 to May 2022 in Shanghai Children's Medical Center were recruited. Children with serious arrhythmia or other conditions such as taking medicine which affects the ventricular function were excluded. The exclusion criteria were poor image quality that was inadequate for analysis. All the children had conventional two-dimensional echocardiography, three-dimensional speckle tracking echocardiography (3DSTE), and CMR examination. In addition, 3DSTE examination was performed on 15 age- and gender-matched children (Control group), who had echocardiography examination for heart murmur but no positive finding. Characteristics of the subjects are shown in Table 1. Informed consent was obtained from parents of these children. The study was implemented according to the standards of the Declaration of Helsinki. The study was approved by the local institutional review board (IRB) and regional research ethics committee (REC) of the Shanghai Children's Medical Center Affiliated to Shanghai Jiao Tong University School of Medicine.

**Table 1:** Characteristics of subjects in this study.

	N	SLV	TA	*p* (N vs. SLV)	*p* (N vs. TA)	*p* (SLV vs. TA)
A (m)	6.600 ± 4.014	7.000 ± 3.380	6.533 ± 4.033	0.956	0.999	0.940
H (cm)	64.867 ± 9.296	64.667 ± 6.789	63.333 ± 8.981	0.998	0.873	0.902
W (kg)	6.473 ± 1.797	6.937 ± 1.642	6.260 ± 1.812	0.751	0.941	0.545
BSA (m^2^)	0.345 ± 0.072	0.357 ± 0.059	0.334 ± 0.071	0.878	0.890	0.615
HR (bpm)	106.533 ± 17.844	119.400 ± 28.053	116.200 ± 21.805	0.285	0.487	0.923
SBP (mmHg)	82.067 ± 6.595	77.867 ± 3.204	76.800 ± 4.784	0.033	0.030	0.517
DBP (mmHg)	54.733 ± 5.535	49.867 ± 3.441	50.400 ± 4.290	0.013	0.025	0.850

A, age; H, height; W, weight; HR, heart rate; BSA, body surface area; SLV, single left ventricle; TA, tricuspid atresia; N: normal children.

### Images acquisition and analysis of echocardiography

Each of the children lay quietly, and oral chloral hydrate (50 mg/kg) was administered if necessary. Children with dextrocardia were scanned in the right decubitus position while the remaining subjects were scanned in the left lateral decubitus position, and the ECG was recorded simultaneously. The peak velocity of the early filling wave (E wave), deceleration time of the E wave (DecT) were measured by two-dimensional echocardiography. The peak velocity of systolic wave (sL) and early filling wave (eL) of left lateral wall, systolic wave (sR) and early filling wave (eR) of right lateral wall, isovolumic relaxation time (IRT), and isovolumetric contraction time (ICT) were measured by tissue Doppler imaging (TDI). MAPSE was defined as mitral annular plane systolic excursion or left lateral annular plane systolic excursion. TAPSE was defined as tricuspid annular plane systolic excursion or right lateral annular plane systolic excursion. The myocardial performance index (MPI), also called the Tei index, is widely used as a good parameter in the assessment of cardiac function in LV as well as single ventricle. This index is calculated by the ratio of total ICT and IRT to ejection time (13); it can be calculated by (*a-b*)/*b*, while “*a*” is the period starting from the end of mitral or atrioventricular valve inflow to the start of next one, and “*b*” is the ejection time of aortic valve. Corresponding parameters of the atrioventricular valve in the SLV group were compared with those of the mitral valve, while average values were used in single left ventricle with two atrioventricular valves. The ratio of E/e was calculated.

**Figure 1:**
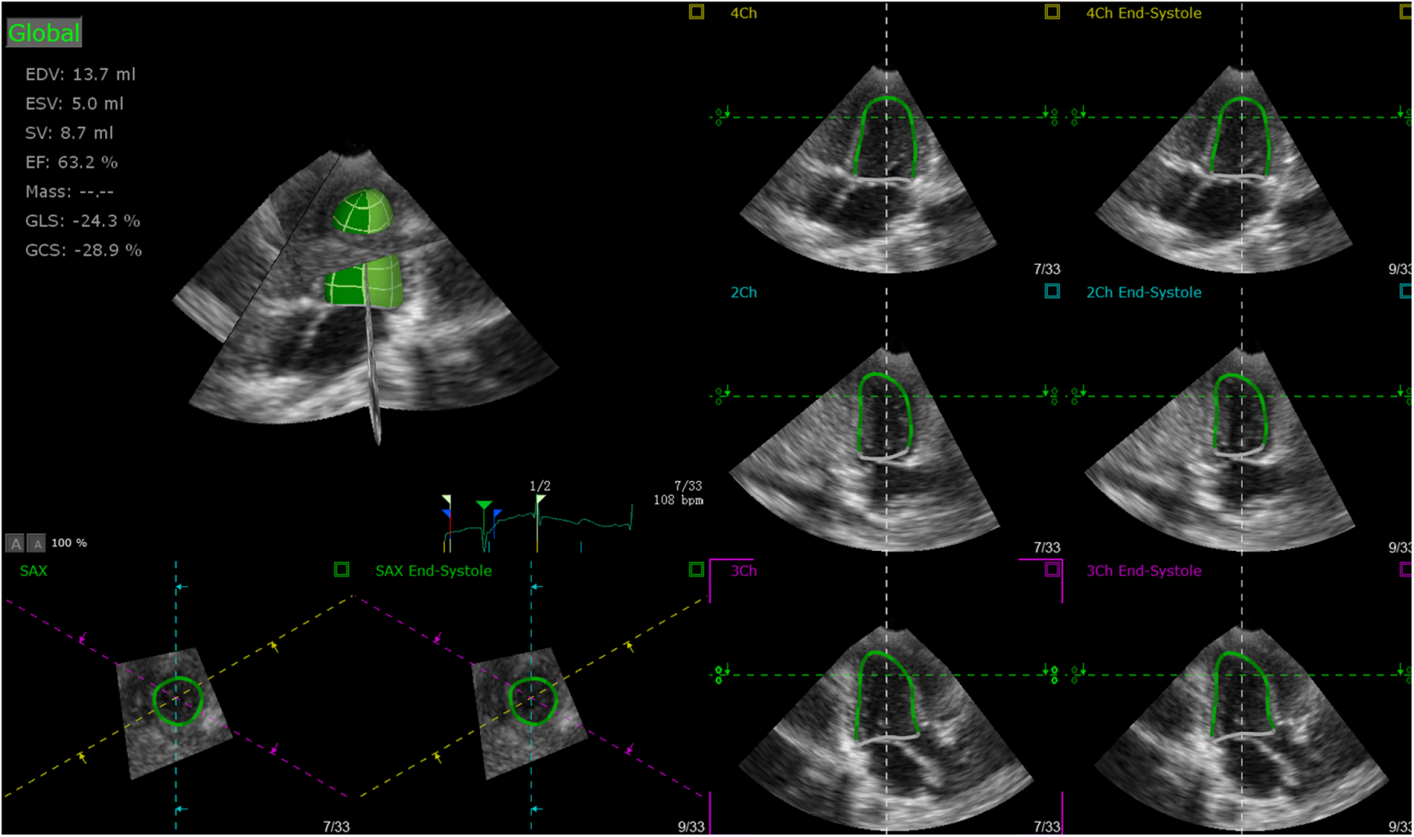
Primary analysis performed by manual border tracking in three planes (two-chamber, three-chamber, and four-chamber).

A Philips iE33 ultrasonic diagnostic apparatus (Philips, Andover, MA, United States), equipped with matrix-array X5-1 transducer was applied to acquire full volume image loops (14). The 3D full volume images were acquired in the apical four-chamber view with its temporal resolution over 20 frames per second and four cardiac cycles per capture, while the 2D images were acquired with its temporal resolution over 55 frames per second. The image loops were stored in a hard drive for further analysis. The clinical characteristics such as age, height, weight, and heart rate (HR) were also collected, and sphericity index (SI) were calculated as follows: SI = *D*_LV_/*L*_LV_, where *D*_LV_ is the diameter of the left ventricle and *L*_LV_ is the length of the left ventricle. TomTec 4D LV-Analysis 3 (Tomtec Imaging Systems GMBH, Unterschleißheim, Germany) was applied for post-processing analysis (15). Primary analysis were performed by manual border tracking in three planes (two-chamber, three-chamber, and four-chamber planes) (Figure 1) and then end diastolic volume (EDV), end systolic volume (ESV), stroke volume (SV), ejection fraction (EF), global longitudinal strain (GLS), global circumstantial strain (GCS), twist, torsion, apical rotation (Rotation_A_), basal rotation (Rotation_B_), average principal tangential strain (TSaverage), average circumferential strain (CSaverage), average longitudinal strain (LSaverage), and average radial strain (RSaverage) were automatically calculated by the software. Images that were not satisfied with automatic tracking could be manually modified and analyzed again.

### Image acquisition and analysis of CMR

CMR studies were performed on a 1.5-T Intera Achieva scanner18 (Philips Medical Systems, Best, The Netherlands) on all the patients. Retrospectively, EKG-gated balanced steady-state free precession (SSFP) cine movies were used. Slice thickness was 4–8 mm, while the interslice gap was set to zero. The temporal resolution was over 30 frames per cardiac cycle. Then images acquired were analyzed using commercial software (Philips View Forum, Andover, MA, United States).

### Reconstruction and computation

Four-dimensional ECHO images were imported into medical imaging software (Materialise®-Mimics 19.0, Plymouth, MI, United States); then, we used the “Thresholding” tool to determine the appropriate gray value range to include all the left ventricle information. Then, we entered the “Edit masks” module, used the “Region growing” and “Calculate 3D from mask” tool to generate the mask and 3D model. At last, the edge was smoothed by the “Smooth mask” tool and smoothed papillary muscles and valves out. After that, the anatomy was represented by five body-fitted prism layers using the commercial software ANSYS ® -ICEM 14.5; velocity obtained by two-dimensional Doppler was used as boundaries for numerical solutions of the Navier–Stokes flow equations. The distance from the first layer to the model surface was 0.02 mm, with 1,000,000 to 3,000,000 tetrahedral mesh elements filling the remaining of the calculated domain, with a tetrahedral mesh filling the remainder of the calculated domain. The blood in the LV is assumed to be an incompressible Newtonian fluid with a constant viscosity of 0.004 kg/ms and density of 1,060 kg/m^3^. The second-order upwind scheme was employed to complete the steady-state numerical simulation by ANSYS®-ICEM 17.0 software. The standard *k* – *ε* model was employed to solve the motion of intraventricular blood flow inside the ventricle. The results were analyzed and processed by ANSYS CFD-Post 14.5 software.

### Statistical analysis

Statistical Package for Social Sciences (SPSS 17.0, SPSS Inc., Chicago, IL, United States) was applied for statistical analysis. Quantitative variables were presented as mean ± SD and compared with *t*-test and Mann–Whitney test between groups. A two-sided *p*-value <0.05 was considered statistically significant. The intraclass correlations (ICCs) (16) of intraobserver and interobserver variability were used to test the reproducibility of 3DSTE. Bland–Altman analysis was used to access the bias and 95% limits of agreement for EDV, ESV, SV, and EF between 3DSTE and CMR. Pearson's correlation coefficient was used to access the correlation between 3DSTE parameters and EF derived from 3DSTE.

## Results

Fifteen patients with SLV (SLV group) and 15 patients with TA (TA group) underwent echocardiography examination. There were no missing data during the study. In addition, 3DSTE examination was performed on 15 age- and gender-matched children (Control group). Then, statistical analysis was performed between patients and controls. Concomitant malformation in SLV group and TA group data is shown in Table 2. Fifteen patients with SLV (100%) and 15 with TA (100%) have ventricular septum defect (VSD). Seven with SLV (46.7%) and 14 with TA (93.3%) have atrial septal defect (ASD), while the one who did not have ASD had single atrium. Five with SLV (33.3%) and five with TA (33.3%) have pulmonary stenosis (PS). Five with SLV (33.3%) and three with TA (20%) have pulmonary hypertension (PH). Twelve of the SLVs have transposition of great artery (TGA) (80%). More detail can be seen in Table 2.

**Table 2:** Concomitant malformation in the SLV and TA groups.

	VSD	ASD/PFO	PS	PH	TGA	PDA	PA	Dextrocardia	Others
SLV1	+	+			+				
SLV2	+		+		+				
SLV3	+			+	+				Moderate atrioventricular regurgitation, Bilateral superior vena cava
SLV4	+		+			+			Atrioventricular valve stenosis
SLV5	+			+	+	+			IAA
SLV6	+			+	+				Atrioventricular valve stenosis
SLV7	+	+	+		+			+	
SLV8	+	+	+		+				
SLV9	+	+				+	+	+	Cor triatriatum sinister
SLV10	+	+			+	+			
SLV11	+				+				Bilateral superior vena cava
SLV12	+			+	+	+			AVSD, moderate atrioventricular regurgitation
SLV13	+	+	+		+	+			
SLV14	+	+		+	+	+			
SLV15	+					+	+		
TA1	+	+				+			
TA2	+	+	+			+			
TA3	+	+		+					
TA4	+	+				+			
TA5	+	+	+						Right aortic arch
TA6	+	+		+					SA, TAPVD
TA7	+	+				+			RVOTO, persistent left superior vena cava
TA8	+	+	+						
TA9	+	+							Persistent left superior vena cava
TA10	+	+					+		
TA11	+	+		+					
TA12	+	+	+						
TA13	+						+		SA, aorta originates from the right ventricle
TA14	+	+				+	+		
TA15	+	+	+			+			Right aortic arch

ASD, atrial septal defect; AVSD, atrioventricular septal defect; IAA, interruption of aortic arch; PA, pulmonary atresia; PDA, patent ductus arteriosus; PFO, patent foramen ovale; PS, pulmonary stenosis; PH, pulmonary hypertension; RVOTO, right ventricular outflow tract obstruction; SA, single atrium; SLV, single left ventricle; TA, tricuspid atresia; TAPVD, total anomalous pulmonary venous drainage; TGA, transposition of great artery; VSD, ventricular septal defect.

**Figure 2:**
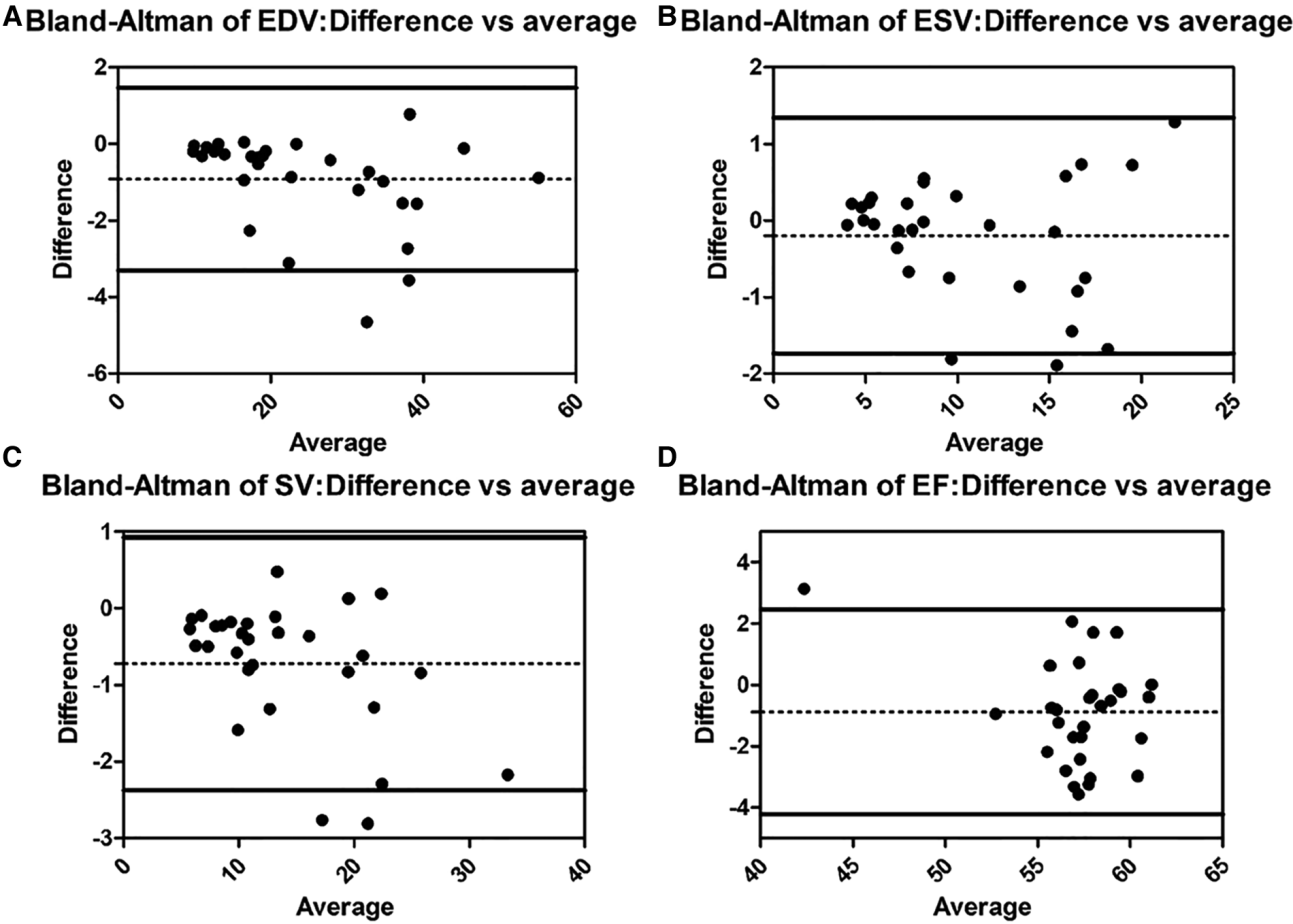
Bland–Altman analysis for EDV, ESV, SV, and EF between 3DSTE and CMR (**A**, Bland–Altman analysis of EDV; **B**, Bland–Altman analysis of ESV; **C**, Bland–Altman analysis of SV; **D**, Bland–Altman analysis of EF). EDV, end diastolic volume; ESV, end systolic volume; SV, stroke volume; EF, ejection fraction; 3DSTE, three-dimensional speckle tracking echocardiography; CMR, cardiac magnetic resonance.

There are no significant differences in age, height, weight, body surface area (BSA), and HR among the three groups (Table 1). Diastolic blood pressure (DBP) and systolic blood pressure (SBP) are lower in both SLV and TA groups than those in the Control group (*p* < 0.05), but they are all within the normal range. The Bland–Altman plot revealed almost no bias between EDV, ESV, SV, and EF measured by 3DSTE and CMR (Figure 2).

### 2D echocardiography

As is shown in Table 3, there are no significant differences in E, sL, eL, E/eL, eR, ICT, and MAPSE between the SLV and the Control groups. While sR and TAPSE are poorer in the SLV group (*p* < 0.05 for sR, *p* < 0.01 for TAPSE), IRT, DecT, and MPI are larger in the SLV than in the Control group (*p* < 0.01). There are no significant differences in E, eL, E/eL, sR, ICT, DecT, and MAPSE between the TA group and the Control group. eR and TAPSE are smaller in the TA group than those in the Control group (*p* < 0.05 for eRv, *p* < 0.01 for TAPSE). sL, IRT, and MPI are larger in the TA group than in the Control group (*p* < 0.01 for sL, *p* < 0.05 for IRT and MPI). There are no significant differences in E, eL, E/eL, sR, eR, IRT, ICT, DecT, TAPSE, and MAPSE between the SLV and the TA groups, while MPI is larger in the SLV group (*p* < 0.05) and sL is larger in the TA Group (*p* < 0.05).

**Table 3:** Parameters of left ventricular function in patients and controls.

	N	SLV	TA	*p* (N vs. SLV)	*p* (N vs. TA)	*p* (SLV vs. TA)
SI	0.667 ± 0.031	0.960 ± 0.169	0.871 ± 0.122	0.000	0.000	0.074
EDV (ml)	12.951 ± 2.384	27.609 ± 12.456	21.089 ± 10.350	0.000	0.000	0.130
ESV (ml)	4.506 ± 1.027	12.280 ± 5.525	8.911 ± 4.479	0.000	0.000	0.065
SV (ml)	8.444 ± 1.487	15.329 ± 7.144	12.178 ± 5.913	0.000	0.005	0.206
EF (%)	65.389 ± 3.119	55.626 ± 3.696	57.887 ± 2.112	0.000	0.000	0.117
GLS (%)	−28.349 ± 2.625	−22.950 ± 3.527	−25.081 ± 3.311	0.000	0.020	0.170
GCS (%)	−29.537 ± 3.934	−23.030 ± 4.154	−25.304 ± 3.876	0.000	0.016	0.274
Twist (°)	12.615 ± 1.569	5.269 ± 2.342	7.603 ± 1.930	0.000	0.000	0.006
Torsion (°/cm)	3.273 ± 0.374	1.437 ± 0.712	2.016 ± 0.557	0.000	0.000	0.020
Rotation_A_ (°)	5.470 ± 1.583	1.740 ± 2.139	3.547 ± 1.864	0.000	0.020	0.031
Rotation_B_ (°)	−7.145 ± 1.052	−3.529 ± 1.821	−4.055 ± 1.564	0.000	0.000	0.061
TSaverage (%)	−38.270 ± 2.237	−30.701 ± 4.326	−31.913 ± 5.276	0.000	0.000	0.431
CSaverage (%)	−29.441 ± 4.133	−22.857 ± 2.847	−25.240 ± 2.923	0.000	0.004	0.139
LSaverage (%)	−27.153 ± 2.051	−23.113 ± 2.843	−24.818 ± 2.449	0.000	0.034	0.154
RSaverage (%)	46.758 ± 2.891	39.362 ± 3.887	40.545 ± 3.647	0.000	0.000	0.627
E	97.713±9.332	89.907±29.144	93.520±24.022	0.332	0.534	0.714
sL	6.115 ± 0.798	6.293 ± 1.729	7.463 ± 2.113	0.678	0.165	0.038
eL	10.161 ± 2.081	10.200 ± 5.251	11.548 ± 4.794	0.604	0.612	0.384
E/eL	9.670 ± 2.045	12.055 ± 6.373	9.553 ± 3.860	0.633	0.627	0.395
sR	10.658 ± 1.962	8.141 ± 4.016	9.225 ± 1.995	0.049	0.356	0.550
eR	14.080 ± 2.154	11.605 ± 5.846	10.233 ± 4.825	0.011	0.008	0.575
IRT	68.200 ± 12.318	81.667 ± 12.726	79.067 ± 9.035	0.007	0.234	0.810
ICT	68.600 ± 14.613	66.533 ± 18.063	66.800 ± 13.154	0.929	0.945	0.999
AduT	95.867 ± 18.267	121.111 ± 20.811	115.444 ± 19.882	0.012	0.059	0.811
DecT	77.867 ± 17.824	101.333 ± 20.486	93.867 ± 27.790	0.017	0.137	0.636
TAPSE	16.433 ± 4.370	10.981 ± 2.766	10.743 ± 2.660	0.000	0.000	0.979
MAPSE	10.239 ± 1.556	9.386 ± 2.394	9.846 ± 2.434	0.287	0.622	0.564
MPI	0.309 ± 0.048	0.477 ± 0.108	0.390 ± 0.105	0.000	0.051	0.034

SI, sphericity index; EDV, end diastolic volume; ESV, end systolic volume; EF, ejection fraction; GLS, global longitudinal strain; GCS, global circumstantial strain; Rotation_A_, apical rotation; MPI, myocardial performance index; Rotation_B_, basal rotation; TSaverage, average principal tangential strain; CSaverage, average circumferential strain; LSaverage, average longitudinal strain; RSaverage, average radial strain; SV, stroke volume; IRT, isovolumic relaxation time; DecT, deceleration time of mitral early filling wave; TAPSE, tricuspid annular plane systolic excursion; MAPSE, mitral annular plane systolic excursion; E: peak velocity of early filling wave of mitral inflow; sL: peak velocity of systolic wave in left lateral wall by tissue doppler imaging, eL: peak velocity of early filling wave in left lateral wall by tissue doppler imaging; sR: peak velocity of systolic wave in right lateral wall by tissue doppler imaging; eR: peak velocity of early filing wave in right lateral wall by tissue doppler imaging; ICT: isovolumic contraction time, AduT: duration time of A wave.

### 3DSTE

**Figure 3:**
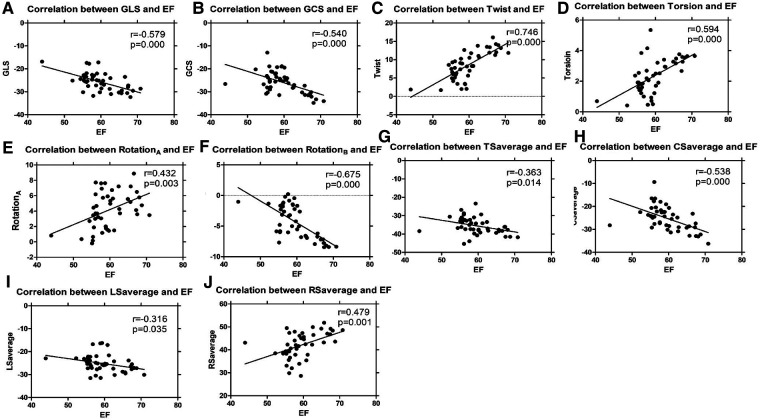
Linear regression analyses of 3DSTE parameters and EF by 3DSTE (**A**, GLS vs. EF; **B**, GCS vs. EF; **C**, Twist vs. EF; **D**, Torsion vs. EF; **E**, Rotation_A_ vs. EF; **F**, Rotation_B_ vs. EF; **G**, TSaverage vs. EF; **H**, CSaverage vs. EF, **I**, LSaverage vs. EF; **J**, RSaverage vs. EF). 3DSTE, three-dimensional speckle tracking echocardiography; EF, ejection fraction; GLS, global longitudinal strain; GCS, global circumstantial strain; RotationA, apical rotation; RotationB, basal rotation; TSaverage, average principal tangential strain; CSaverage, average circumferential strain; LSaverage, average longitudinal strain; RSaverage, average radial strain.

The ICCs of 3DSTE in 45 children are shown in Table 4. The intraobserver and interobserver variability are good. Intraobserver and interobserver variations are within the clinically acceptable ranges, confirming the repeatability of 3DSTE. Our results show a good consistency between 3DSTE and CMR (Figure 2), and the intraclass correlation of EDV, ESV, SV, and EF between 3DSTE and CMR are 0.995, 0.989, 0.992, and 0.879, respectively. The correlation between EF derived from 3DSTE and other parameters are shown in Figure 3. The r value of GLS, GCS, twist, torsion, Rotation_A_, Rotation_B_, TSaverage, CSaverage, LSaverage, and RSaverage are −0.579, −0.540, 0.746, 0.594, 0.432, −0.675, −0.363, −0.538, −0.316, and 0.479, respectively. Twist is best correlated with EF measured by 3DSTE.

**Table 4:** Intraobserver and interobserver variations of 3DSTE.

	Intraobserver variability		Interobserver variability	
	ICC	*p*	ICC	*p*
EDV	0.988 (0.977–0.993)	0.000	0.962 (0.932–0.979)	0.000
ESV	0.982 (0.968–0.990)	0.000	0.960 (0.928–0.978)	0.000
SV	0.977 (0.959–0.987)	0.000	0.956 (0.921–0.976)	0.000
EF	0.771 (0.618–0.867)	0.000	0.824 (0.702–0.900)	0.000
GLS	0.828 (0.708–0.902)	0.000	0.767 (0.613–0.865)	0.017
GCS	0.808 (0.677–0.890)	0.000	0.808 (0.643–0.901)	0.000
Twist	0.884 (0.799–0.935)	0.000	0.871 (0.778–0.927)	0.000
Torsion	0.800 (0.663–0.885)	0.000	0.827 (0.706–0.901)	0.000
Rotation_A_	0.984 (0.967–0.992)	0.000	0.862 (0.762–0.921)	0.000
Rotation_B_	0.798 (0.661–0.884)	0.000	0.716 (0.537–0.833)	0.000
TSaverage	0.783 (0.638–0.875)	0.000	0.784 (0.639–0.875)	0.000
CSaverage	0.835 (0.719–0.906)	0.000	0.799 (0.662–0.884)	0.000
LSaverage	0.757 (0.597–0.859)	0.000	0.814 (0.686–0.893)	0.000
RSaverage	0.850 (0.743–0.915)	0.000	0.785 (0.641–0.876)	0.000

3DSTE, three-dimensional speckle tracking echocardiography; EDV, end diastolic volume; ESV, end systolic volume; EF, ejection fraction; GLS, global longitudinal strain; GCS, global circumstantial strain; Rotation_A_, apical rotation; Rotation_B_, basal rotation; TSaverage, average principal tangential strain; CSaverage, average circumferential strain; LSaverage, average longitudinal strain; RSaverage, average radial strain; ICC, intraclass correlation coefficient.

As shown in Table 3, SI, EDV, ESV, and SV are larger, and EF, GLS, GCS, twist, torsion, Rotation_A_, Rotation_B_, TSaverage, CSaverage, LSaverage, and RSaverage are poorer in the SLV group (*p* < 0.01) than those in the Control group. SI, EDV, ESV, and SV are larger, and EF, GLS, GCS, Twist, Torsion, RotationA, RotationB, TSaverage, CSaverage, LSaverage, and RSaverage are poorer in the TA group than those in the Control group (p < 0.05 for GLS, GCS, RotationA, and LSaverage, p < 0.01 for others). The differences in SI, EDV, ESV, SV, EF, GLS, GCS, TSaverage, LSaverage, and RSaverage are not significant between the SLV group and the TA group. However, twist, torsion, and RotationA are poorer in SLV Group (p < 0.01 for Twist, p < 0.05 for Torsion and RotationA).

### CFD

**Figure 4:**
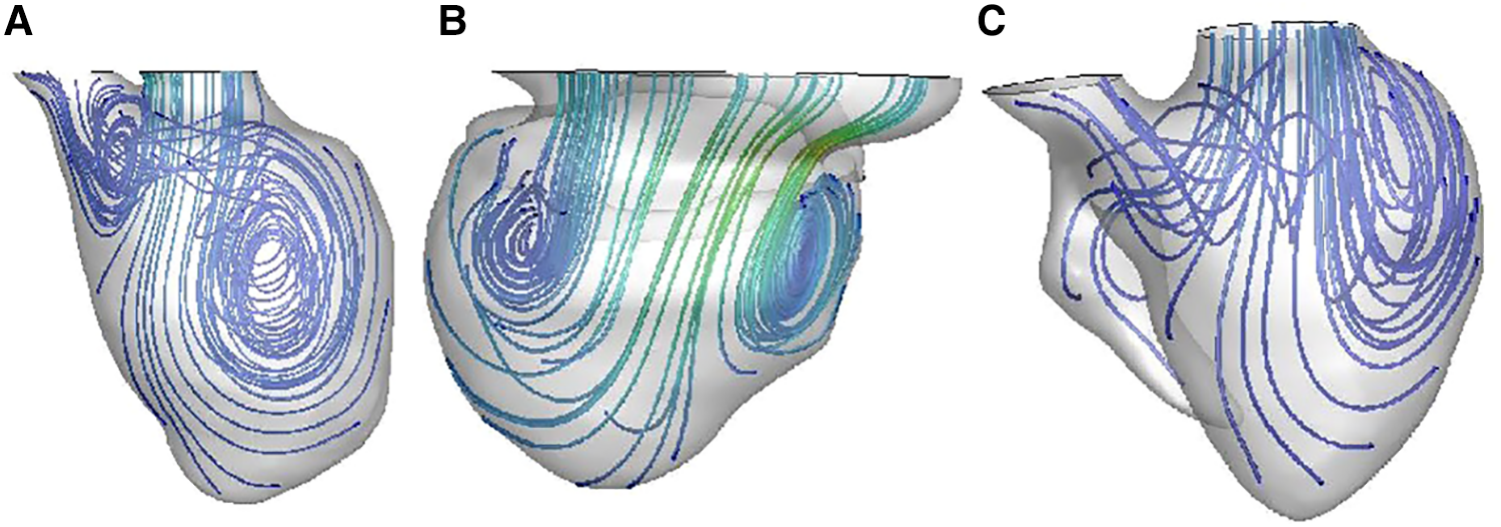
Blood streamlines in ventricular chamber in normal heart (**A**), patient with SLV (**B**), and patient with TA (**C**).

**Figure 5:**
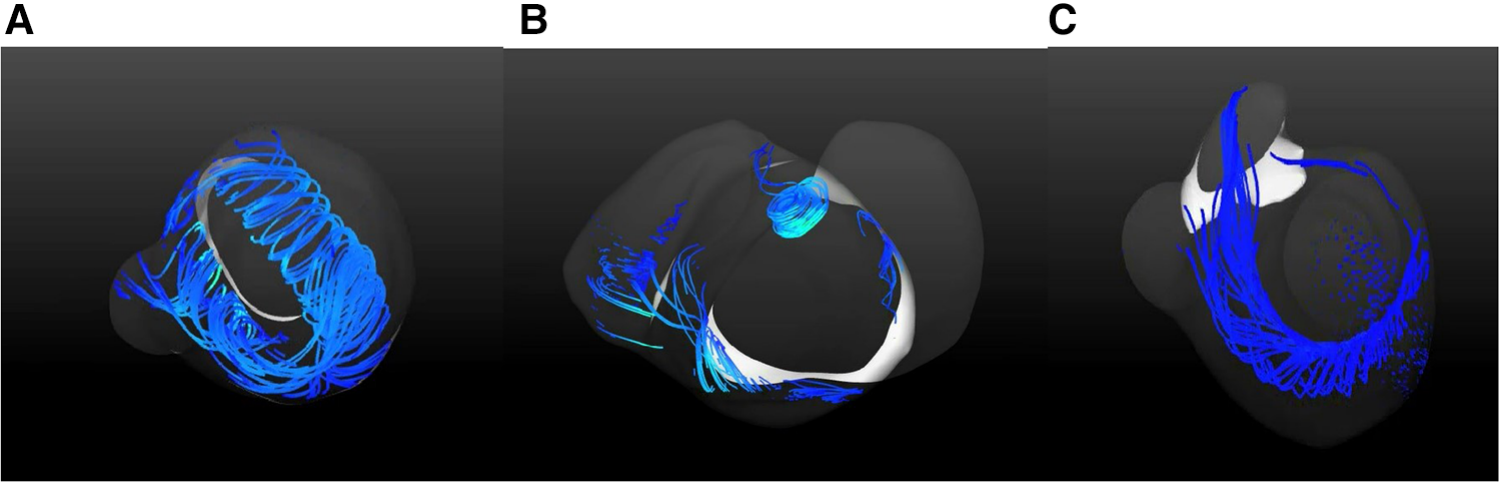
Vortex rings in LV in normal heart (**A**), SLV (**B**) and TA (**C**).

As shown in Figures 4, 5, vortexes differ a lot among the three groups. In normal left ventricle, during diastolic period, blood flows through the mitral valve and goes forward until reaching the apex, and then it swirls and goes backward, forming a major vortex in the middle of the left ventricle, with a counterclockwise rotation. After that, a vortex ring is formed in the base region of LV, and then, blood goes to the outflow tract region and forms a small clockwise rotation vortex. This is a consecutive and interactive process. In patients with SLV, blood flows straight ahead to the apex, and it spreads out in a fan-shaped manner and form two small vortexes. When viewed from the front, it is clockwise on the left and counterclockwise on the right in the base region of the LV. The vortex ring during the diastolic phase is incomplete. In the TA group, the main vortex is similar to the one in the normal LV chamber, but smaller, and its center is closer to the apex and the lateral side. The vortex ring during diastolic phase is incomplete.

## Discussion

In patients with SLV and TA, abnormal systolic and diastolic function are common, but they are usually evaluated separately. A previous study had implied that impaired relaxation may result from decreased systolic motion (17). In reality, systolic function and diastolic function are closely interrelated, while contractility is impaired; elastic recoil in early diastole may be also impaired too. Systolic function contributes to diastolic function through restoring forces or diastolic recoil of the myocardium from tension produced in systole. Energy stores during systolic twisting and releases during diastolic untwisting; therefore, ventricular relaxation and early diastolic filling are finished. On the other hand, since ejection depends a lot on enough filling during diastole through the Frank–Starling mechanism, diastolic function also affects contraction. Actually, both systolic and diastolic function are impaired before surgery in patients with FSLV.

### Similarities between the SLV group and the TA group

#### 2D echocardiography

There are no significant difference in E/eL ratios among these three groups, similar to the study by Hershenson et al. (18), indicating that end diastolic pressures are not increased in SLVs or TAs; some other studies also show weak correlation between E/e and filling pressures (19). Although the E/eL ratio is a common parameter to evaluate diastolic function in adults, it may be less valuable in children as it reflects elevated filling pressures, which may be less common in children (20). In our study, relatively younger age and relatively normal left lateral wall motion, resulting to relatively normal MAPSE.

Patients in both SLV and TA groups have prolonged IRT than those in the Control group. Prolongation of isovolumic time is likely to be affected by the slow rate of ventricular relaxation resulting from decline in the change rate of ventricular pressure or decrease in ventricular compliance. Such changes are likely correlated to abnormal myocardial untwisting. As presented by Appleton et al. (21), longer IRT is associated with impaired relaxation and normal filling pressures, and is the earliest and commonest change seen with diastolic abnormality.

Patients with FSLV have higher MPI and lower TAPSE values than those in the Control group, similar to previous studies (22, 23). MPI depends on events during isovolumetric contraction, ejection, and isovolumetric relaxation, and its increase reflects impaired systolic and/or diastolic function. The decrease of TAPSE reflects impaired myocardial motion of the right lateral wall.

#### 3DSTE

Patients in the SLV and TA groups had enlarged EDV, ESV, and SV, which are consistent with previously reports (10). SLV and TA have shared characteristics: the domination of left ventricle and hypoplastic right ventricle. A previous study on angiography reported that LV dysfunction was a consequence of longstanding LV volume overload (24). LVs in both TA and SLV are overloaded because of the hypogenesis of RV; therefore, the left ventricles have to bear much more work than they usually do. The ventricular volume increases as a response aiming to maintain the normal cardiac output. Initially, though patients with SLV and TA have abnormal relaxation and impaired systolic function, EF is preserved. Subsequently, these adaptive mechanisms become insufficient, and the ventricle gradually evolves into decompensation, and at last, evident global pump dysfunction.

An experimental study (25) showed the volume overload could increase the collagen deposition in the pressure-overloaded ventricle, causing myocardial stiffness. On histologic examination, the gross pathologic change of increased LV volume is considered to be driven by the cardiomyocyte hypertrophy and apoptosis as well as the increased interstitial collagen (26).

Not surprisingly, strain values in FSLV children are significantly poorer than those in the Control group, which are consistent with previous reports (27). In normal ventricle, the myofibers are oriented in three broad layers: the superficial fibers are oriented obliquely, the middle fiber circumferentially, and the deep layer runs longitudinally (28). Myocardium contracts and relaxes with the fiber shortening and extension parallel to its orientation. While in the case of FSLV, fibers are “irregular” (29), the irregular orientation of subepicardial fibers may lead to lower circumferential strain and radial strain (30). The lower value of strain in FSLV patients can indicate nonischemic fibrosis, which is validated by late gadolinium enhancement and histologic findings (31, 32).

Myocardial fibrosis in FSLV is also an underlying reason for its impaired cardiac function, which is confirmed by cardiac magnetic resonance imaging (33). Myocardial fibrosis is intrinsically linked to LV stiffness and chamber modification (34) and is a common final pathway in chronic myocardial disease and is the structural correlate of heart failure (35). At the histological level, the myocardium of SLV has been shown to be more fibrotic than normal (36). This may explain, at least partially, the clinical observation of the abnormal performance at an early age of FSLV.

Our study also showed poorer twist, torsion, apical, and basal rotation in children with FSLV when compared with controls, as previous reported (11, 37), and may be reflective of their different fiber orientations. Rotation, torsion, and twist play an important role in both systolic and diastolic function; they distribute stress and energy across the ventricle and enhance ventricular filling by restoring potential energy and diastolic elastic recoil during ejection. Twist is the net difference in rotation of the LV apex and base and plays an important role in LV mechanics (38). Untwist, the subsequent recoil of twist, can release the restored forces, impacting LV diastolic relaxation and early diastolic filling; these may be early signs of cardiac dysfunction before clinical symptom.

#### CFD

In a normal left ventricle, myocardial relaxation and contraction push flood flow with gyratory motion. The geometry and myofiber orientation of a normal left ventricle are optimized to eject blood in the systole with least energy dissipation and stored potential energy for untwisting; by untwisting, blood is effectively sucked into the ventricle. Recoiling during diastole allows the myocardium to relax and suck blood from left atrium into left ventricle, with vortexes and vortex rings formed in LV, which can redirect the flow and preserve energy. It can provide dynamic energy sources for blood to flow forward, forming vortexes, which can convert vortex kinetic energy into the rotational kinetic energy, and avert convective deceleration. Natural asymmetric geometry of the heart could minimize dissipative interaction of flow convection to arrange the flow for ejection.

While in patients with FSLV vortexes in ventricular chambers are smaller and rounder, and even disappear, the vortex ring during the diastolic phase is incomplete. The enlarged dominated chamber, hypoplastic residual cavity and the symmetric geometry make the structure of FSLV significantly different from normal LV; therefore, the streamline inside the ventricle is disordered and flows without enough swirling, resulting in smaller vortex and incomplete vortex ring. Thus, more energy dissipates, and more performance is needed to maintain proper stroke volume.

Therefore, the abnormal orientation of the myofiber, fibrosis of myocardium, special geometry, and disordered flow fields, along with the overload of the LV, may cause weakness of contractility in systole as well as diastole, deteriorating the ventricular performance.

### Differences between the SLV group and the TA group

#### 2D echocardiography

It is notable that sL is larger in the TA group compared with the SLV group, and even the Control Group, indicating that the left lateral wall in the TA group contributes more to cardiac function, which may reflect adaptation and compensation; it may also explain the better performance in the TA group than in the SLV group. Patients in the TA group also had higher sR value than those in the SLV group, indicating that cardiac motion in the right lateral wall in the TA group are better than the SLV group, similar to a previous study (30).

Compared with the Control group, patients in the SLV group have more prolonged DecT; the difference is significant, while the difference between the TA group and the Control group is not significant, reflecting decreased ventricular compliance in early diastolic period in SLV than in TA. As stated by Thomas and Weyman (39), impairment of LV relaxation results in the prolongation of IRT, DecT, and lower E.

Though without significant difference, there is a trend toward lower values of E in the SLV group than the TA and Control groups. Lower E is suggestive of volume overload and increased atrial reliance during diastolic in TA for ventricular filling, similar to the study by Khoo et al. (40). This increased contribution on atrial active function is a compensatory of ventricular diastolic dysfunction. Different from a normal atrium, atrial contractility plays an important role in these patients and acts as a contributor to more passive conduit properties. One possibility for this may be an indicator of the abnormal diastolic properties as previously mentioned (41) and the compensatory function of the atrium. This has been clearly demonstrated in adult studies where atrial properties are affected by ventricular diastolic abnormalities (42).

#### 3DSTE

Compared with the TA group, patients in the SLV group have poorer twist, torsion, and rotation. The orientation of myofibers is more longitudinal in TA, with interstitial fibrous deposition greater than normal heart in all sites analyzed (43); this was found in the first weeks of life and increases with age, as found in a previous study (44), while the superficial fibers are circumferentially oriented in SLV (45). Though similar in volume overload and more spherical shape than normal children, LVs in TA and SLV have different characteristics; though not significant, SI in the SLV group is larger than the TA group. As shown in Table 3 and Figure 3, twist is well correlated with EF and can help distinguish TA group from SLV group sensitively; therefore, twist may be a good measure for the assessment of ventricular performance.

#### CFD

The shape of the ventricle may affect a lot in the turning of streamlines and the formation of the vortices. Compared with the TA group, LV in the SLV group has rounder apex and more symmetrical structure, while it is more ellipsoidal and asymmetrical in TA. In patients with SLV, blood flows straight ahead to the apex without enough swirling; when the blood flow reaches the enlarged part in the middle of the main cardiac cavity, it spreads out in a fan-shaped manner and forms two small vortices. When viewed from the front, it is clockwise on the left and counterclockwise on the right in the base region of LV. More energy dissipates during this period. The unnatural asymmetry flow could reduce the efficiency of the heart pump by more than 10% (46). In the TA group, the main vortex is similar to the one in a normal LV chamber, but smaller, and its center is closer to the apex and the lateral side. We cautiously speculate that the obtuse apex as well as the more symmetric structure of the dominant chamber in SLV may affect the blood streamline in LV.

### Limitations

Since the A wave fused into the E wave in many children in the study, we did not discuss these parameters in our article. Not only the impairment of cardiac function but also the preload, volume, and atrial and ventricular stiffness influence the results, but it is difficult to distinguish them completely. The shape of the ventricle and anatomic structures such as valve and papillary muscle may affect the turning of streamlines and the formation of the vortices, while in our study, valves and papillary muscles were smoothed out to optimize CFD modeling. Our sample size of this study was relatively small. Furthermore, more patients and long-term follow-up are needed for further study.

## Conclusion

Both SLV and TA have impaired systolic and diastolic function. Patients with SLV have poorer cardiac function than those with TA due to less compensation and more disordered streamline. Our study demonstrates that twist is a good indicator for measuring ventricular performance, which may predate more noticeable echocardiographic signs of deterioration.

## Data Availability

The raw data supporting the conclusions of this article will be made available by the authors, without undue reservation.
