# Corrigendum: Comparison of cardiac function between single left ventricle and tricuspid atresia: assessment using echocardiography combined with computational fluid dynamics

**DOI:** 10.3389/fped.2024.1368440

**Published:** 2024-01-31

**Authors:** Li-Jun Chen, Lan-Ping Wu, Lei-Sheng Zhao, Zhi-Fang Zhang, Jin-Long Liu, Wen-Jing Hong, Shu-Wen Zhong, Sheng-Fang Bao, Jing Yang, Yu-Qi Zhang

**Affiliations:** ^1^ Department of Pediatric Cardiology, Shanghai Children’s Medical Center, School of Medicine, Shanghai Jiao Tong University, Shanghai, China; ^2^ Institute of Pediatric Translational Medicine, Shanghai Children’s Medical Center, School of Medicine, Shanghai Jiao Tong University, Shanghai, China; ^3^ Department of Ultrasound, Jiaxing University Affiliated Women and Children Hospital, Jiaxing, China

**Keywords:** functional single left ventricle, tricuspid atresia, single left ventricle, cardiac function, three-dimensional speckle tracking echocardiography (3DSTE), computational fluid dynamics—CFD

A Corrigendum on Comparison of cardiac function between single left ventricle and tricuspid atresia: assessment using echocardiography combined with computational fluid dynamics By Chen L-J, Wu L-P, Zhao L-S, Zhang Z-F, Liu J-L, Hong W-J, Zhong S-W, Bao S-F, Yang J and Zhang Y-Q (2023). Front. Pediatr. 11:1159342. doi: 10.3389/fped.2023.1159342

**Incorrect Author Name**

In the published article, an author name was incorrectly written as “Jin-Llong Liu”. The correct spelling is “Jin-Long Liu”.

The authors apologize for this error and state that this does not change the scientific conclusions of the article in any way. The original article has been updated.

**Error in the Correspondence**

In the published article, a corresponding author name was incorrectly written as “Yuqi Zhang”. The correct spelling is “Yu-Qi Zhang”.

The authors apologize for this error and state that this does not change the scientific conclusions of the article in any way. The original article has been updated.

